# Association of metabolic syndrome with ruptured status of intracranial aneurysms in a definitively treated cohort: a retrospective cohort analysis

**DOI:** 10.3389/fneur.2026.1817370

**Published:** 2026-05-22

**Authors:** Zisheng Liu, Xidong Wu, Jiaming Xu, Jianyong Cai, Huajun Ba, Qun Lin, Jun Sun, Weizhong Shi

**Affiliations:** 1 Department of Neurosurgery, Wenzhou Central Hospital, Zhejiang, Wenzhou, China; 2 Intervention Center, Wenzhou Central Hospital, Zhejiang, Wenzhou, China

**Keywords:** dyslipidemia, hyperglycemia, hypertension, intracranial aneurysm rupture, metabolic syndrome

## Abstract

**Background:**

Metabolic syndrome (MetS) may contribute to vascular instability through systemic inflammation and endothelial dysfunction. Its association with ruptured intracranial aneurysm (IA) status remains unclear. We examined whether MetS, its burden, and its individual components were associated with ruptured status in a cohort of patients with intracranial aneurysms who underwent definitive treatment, including both ruptured aneurysms and unruptured aneurysms selected for treatment.

**Methods:**

We performed a single-center retrospective cohort analysis of patients with intracranial aneurysms who underwent endovascular treatment at Wenzhou Central Hospital between January 2018 and January 2024, including 484 unruptured and 344 ruptured aneurysms. The cohort included patients with ruptured aneurysms and patients with unruptured aneurysms selected for definitive treatment; conservatively managed unruptured aneurysms were not included. MetS was defined as the presence of at least three standard components. Multivariable logistic regression was used to evaluate associations of binary MetS, MetS score, and individual MetS components with ruptured aneurysm status. Model discrimination was assessed using receiver operating characteristic analysis. A morphology-adjusted sensitivity analysis was performed in a 400-patient imaging subsample.

**Results:**

MetS was more prevalent in the ruptured group than in the unruptured group (59.9% vs. 40.1%). Binary MetS was independently associated with ruptured status (adjusted OR 1.83, 95% CI 1.31–2.54). Higher MetS scores showed a graded association with ruptured status, although estimates were less precise at the highest score categories. Among individual components, elevated glucose (adjusted OR 2.56, 95% CI 1.84–3.56), reduced high-density lipoprotein cholesterol (adjusted OR 2.35, 95% CI 1.62–3.40), and elevated blood pressure (adjusted OR 1.71, 95% CI 1.14–2.55) showed the strongest associations. The component-based model had the highest AUC (0.711), indicating moderate discrimination. The morphology-adjusted sensitivity analysis showed broadly stable results.

**Conclusion:**

In this cohort of patients with intracranial aneurysms undergoing definitive treatment, MetS and greater MetS burden were associated with ruptured aneurysm status. Elevated glucose, reduced high-density lipoprotein cholesterol, and elevated blood pressure showed the strongest associations. These findings should be interpreted as associative rather than causal and require prospective multicenter validation.

## 1 Introduction

Intracranial aneurysm (IA) rupture can precipitate subarachnoid hemorrhage, a catastrophic event with mortality up to 50% (1, 2). The incidence of aneurysmal SAH is relatively low (approximately 9 per 100,000 person-years), but its consequences are often catastrophic – survivors may suffer hydrocephalus, vasospasm, rebleeding, and permanent neurological deficits (2). Unruptured intracranial aneurysms (UIAs) are common (estimated prevalence 2%–3% of the general population), yet only a fraction will rupture during a patient’s lifetime. Identifying which aneurysms are at highest risk of rupture remains a major clinical challenge (3). Existing risk stratification models (e.g., the PHASES score) incorporate factors like aneurysm size, location, hypertension, and patient demographics, but their predictive performance is limited (low specificity and only moderate predictive value) (2, 4). In practice, many patients harboring UIAs face uncertainty because current tools cannot reliably distinguish “ticking time bombs” from aneurysms unlikely to rupture. This clinical uncertainty underscores the need to explore additional clinically accessible factors associated with ruptured aneurysm status and to generate hypotheses for future prospective validation.

Metabolic syndrome (MetS) is a cluster of interrelated metabolic abnormalities, classically including central obesity, elevated blood pressure(BP), impaired glucose regulation, and atherogenic dyslipidemia (high triglycerides and low high-density lipoprotein cholesterol) (5, 6). By definition, the presence of MetS reflects an insulin-resistant, pro-inflammatory state; indeed, MetS results in systemic inflammation and at least doubles the risk of cardiovascular disease (5–7). Mechanistically, the metabolic derangements in MetS lead to endothelial dysfunction and oxidative stress in the vasculature (8). Excess adiposity promotes the release of inflammatory cytokines and adipokines that injure the endothelium and trigger chronic low-grade inflammation (9). Lipid disorders in MetS (e.g., elevated apolipoprotein-B lipoproteins) cause lipid accumulation and peroxidation within the arterial wall, provoking degenerative changes such as smooth muscle cell phenotypic modulation, endothelial cell dysfunction and apoptosis (10). In this way, MetS creates a systemic milieu of inflammation and atherosclerosis that could plausibly affect cerebral arteries and aneurysm stability.

To address this knowledge gap, we conducted a retrospective study in a cohort of patients with intracranial aneurysms who underwent definitive treatment, including patients with ruptured aneurysms and patients with unruptured aneurysms selected for treatment, to examine whether MetS, as a composite condition and through its individual components, was associated with ruptured aneurysm status. Our objectives were to determine whether the presence and burden of MetS were associated with ruptured status, to identify which MetS components showed the strongest associations, to assess whether MetS-related variables provided incremental discriminative information beyond basic clinical factors, and to explore whether these associations varied across selected clinical subgroups. Through this analysis, we aimed to generate.

Hypothesis-supporting evidence for future prospective and multicenter studies rather than to establish causality or a definitive rupture-prediction model. Because conservatively managed unruptured aneurysms were not included, this study was designed to evaluate associations with ruptured status within a treated cohort rather than to represent the full natural-history spectrum of unruptured intracranial aneurysms.

## 2 Methods

### 2.1 Study design and population

We conducted a single-center retrospective analysis of patients who underwent endovascular treatment of IAs at Wenzhou Central Hospital from January 2018 through January 2024. The cohort included patients with ruptured aneurysms and patients with unruptured aneurysms selected for definitive treatment; conservatively managed unruptured aneurysms were not included. The cohort was stratified into two comparative groups: ruptured aneurysms (presenting with subarachnoid hemorrhage) and unruptured aneurysms (incidentally discovered or symptomatic without hemorrhage).

While aneurysm morphometry constitutes a critical determinant of rupture propensity, our investigation was constrained by inherent limitations of retrospective methodology. Specifically, comprehensive volumetric and morphological parameters were not systematically documented across all cases. To address this constraint and enhance between-group comparability, we implemented a rigorous selection approach for the unruptured cohort, including only those patients who underwent definitive intervention based on our institutional protocol (Table 1). This selection strategy ensured that only higher-risk unruptured aneurysms (based on size ≥5 mm, posterior circulation location, irregular morphology, or documented growth) were included for comparison with the ruptured group. Accordingly, our findings should be interpreted as associations within a treated IA cohort rather than estimates generalizable to all unruptured intracranial aneurysms in the community. Because conservatively managed unruptured aneurysms were excluded by design, this cohort does not reflect the natural history spectrum of all unruptured IAs.

**Table 1:** Institutional protocol for management of unruptured intracranial aneurysms.

Characteristic	Risk stratification	Management approach
Size-based criteria	<5 mm, anterior circulation	Conservative management with surveillance imaging
	5–7 mm	Intervention considered with additional risk modifiers
	≥7 mm	Intervention typically recommended
	≥10 mm	Intervention strongly indicated
	≥25 mm (giant)	Urgent intervention warranted
Location-based criteria	Posterior circulation	Lower size threshold for intervention (even <5 mm)
	Basilar apex	High-risk location; intervention generally indicated regardless of size
	Anterior circulation	Size-dependent approach (see above)
Morphological features	Complex configuration	Intervention typically indicated due to wall instability concerns
	Dome-to-neck ratio <2	May necessitate specialized techniques (stent/balloon assistance)
	Progressive enlargement	Intervention indicated regardless of initial dimensions

All treatment decisions undergo multidisciplinary consensus review. Protocol reflects institutional experience and may not be universally applicable.

Study eligibility required: (1) radiographic confirmation of aneurysm diagnosis via CTA, MRA, or DSA; (2) treatment with either endovascular or microsurgical techniques; and (3) complete clinical and laboratory documentation. Exclusion criteria encompassed: (1) incomplete medical records; (2) concomitant cerebrovascular pathologies (arteriovenous malformations, moyamoya arteriopathy); (3) indeterminate rupture source in multi-aneurysm cases; (4) conservatively managed cases; and (5) infectious or inflammatory aneurysm etiology.

### 2.2 Data collection

Data were collected from electronic medical records and included a comprehensive set of demographic, clinical, and laboratory parameters. Demographic data included age, gender, and lifestyle factors such as smoking and alcohol consumption. Clinical data included comorbidities such as hypertension, diabetes mellitus, and coronary artery disease.

In this retrospective study, laboratory and clinical measurements were obtained from each patient’s earliest available hospital records prior to any interventional or surgical treatment. For patients with ruptured aneurysms, blood samples were collected in the emergency department immediately after admission and before definitive treatment. For patients in the unruptured cohort, laboratory data were derived from routine preoperative evaluation, typically within 24 h of admission.

BP measurements were conducted by trained nursing staff using an automated sphygmomanometer, with patients in a seated position after at least 5 min of rest. If multiple BP readings were available, the average of the first two measurements was recorded.

All laboratory assays—including glucose, triglycerides, and HDL cholesterol—were performed in the hospital’s central laboratory, adhering to standardized protocols for calibration and quality control. Wherever possible, fasting blood samples were used to minimize variability due to recent food intake. These measurements represented the earliest available pre-intervention data. However, in the ruptured group, blood pressure and glucose values may still have been influenced by acute post-subarachnoid hemorrhage physiology and therefore should not be interpreted as true pre-rupture long-term metabolic status.

#### 2.2.1 MetS was defined according to the following criteria

BMI ≥ 28 kg/m^2^, Triglycerides (TG) ≥ 1.7 mmol/L (≥150 mg/dL), HDL cholesterol (HDL-C) < 1.0 mmol/L (for males) or < 1.3 mmol/L (for females), Systolic blood pressure (SBP) ≥ 130 mmHg or diastolic blood pressure (DBP) ≥ 85 mmHg (or known hypertension), Glucose ≥ 5.6 mmol/L (100 mg/dL) or known diabetes.

Each patient’s data were marked for the presence or absence of each component. The presence of three or more components of MetS was used to diagnose MetS.

In addition to these clinical parameters, laboratory data, including blood glucose levels, lipids (triglycerides, HDL-C), and BP readings, were also extracted from patient records.

Because comprehensive imaging data were not uniformly available across all patients—particularly among those who underwent emergency surgery for ruptured aneurysms—we were unable to extract standardized aneurysm morphology variables (e.g., exact diameter, dome-to-neck ratio, irregularity scoring) for the full cohort. To evaluate whether ruptured and unruptured aneurysms were reasonably comparable in terms of imaging characteristics, we conducted a dedicated imaging subsample analysis. We randomly selected 400 patients (200 ruptured and 200 unruptured) with complete and retrievable imaging data and extracted maximum diameter, aneurysm location, and morphological features (irregular pattern, bifurcation vs. sidewall configuration, wide-neck morphology, and multiplicity). These variables were compared between groups using standard statistical tests.

As a sensitivity analysis, we further examined the robustness of the main association in the imaging subsample with available aneurysm morphology data (n = 400). Using the same clinically adjusted framework as in the main analysis, we additionally adjusted for aneurysm irregularity, multiple aneurysms, grouped aneurysm location, bifurcation/sidewall type, maximum aneurysm diameter, and neck ratio. To preserve parsimony in this subsample analysis, MetS burden was additionally modeled as an ordinal score per 1-point increase.

### 2.3 Statistical analysis

Descriptive statistics were used to summarize demographic, clinical, and metabolic characteristics. Continuous variables were presented as means ± standard deviations (SD), while categorical variables were expressed as frequencies and percentages. Differences between the ruptured and unruptured aneurysm groups were compared using t-tests for continuous variables and chi-square tests for categorical variables.

Univariate logistic regression was used to assess the association of demographic, clinical, and metabolic factors with ruptured aneurysm status. Variables with a *p*-value < 0.05 in the univariate analysis were included in the multivariate logistic regression models. Three models were evaluated: Model 1: MetS binary classification (presence vs. absence of MetS), Model 2: MetS score (0–5), Model 3: Individual components of MetS (BMI, triglycerides, HDL-C, BP, and glucose).

Receiver operating characteristic (ROC) analysis was used to compare the discrimination of the multivariable models for distinguishing ruptured from unruptured aneurysms. A higher AUC indicates better discrimination between ruptured and unruptured status within this cohort.

Decision curve analysis (DCA) was used as an exploratory assessment of relative net benefit across threshold probabilities. The net benefit was calculated for each model to evaluate its potential for improving clinical decision-making compared to alternative strategies.

## 3 Results

### 3.1 Baseline characteristics

**Figure 1:**
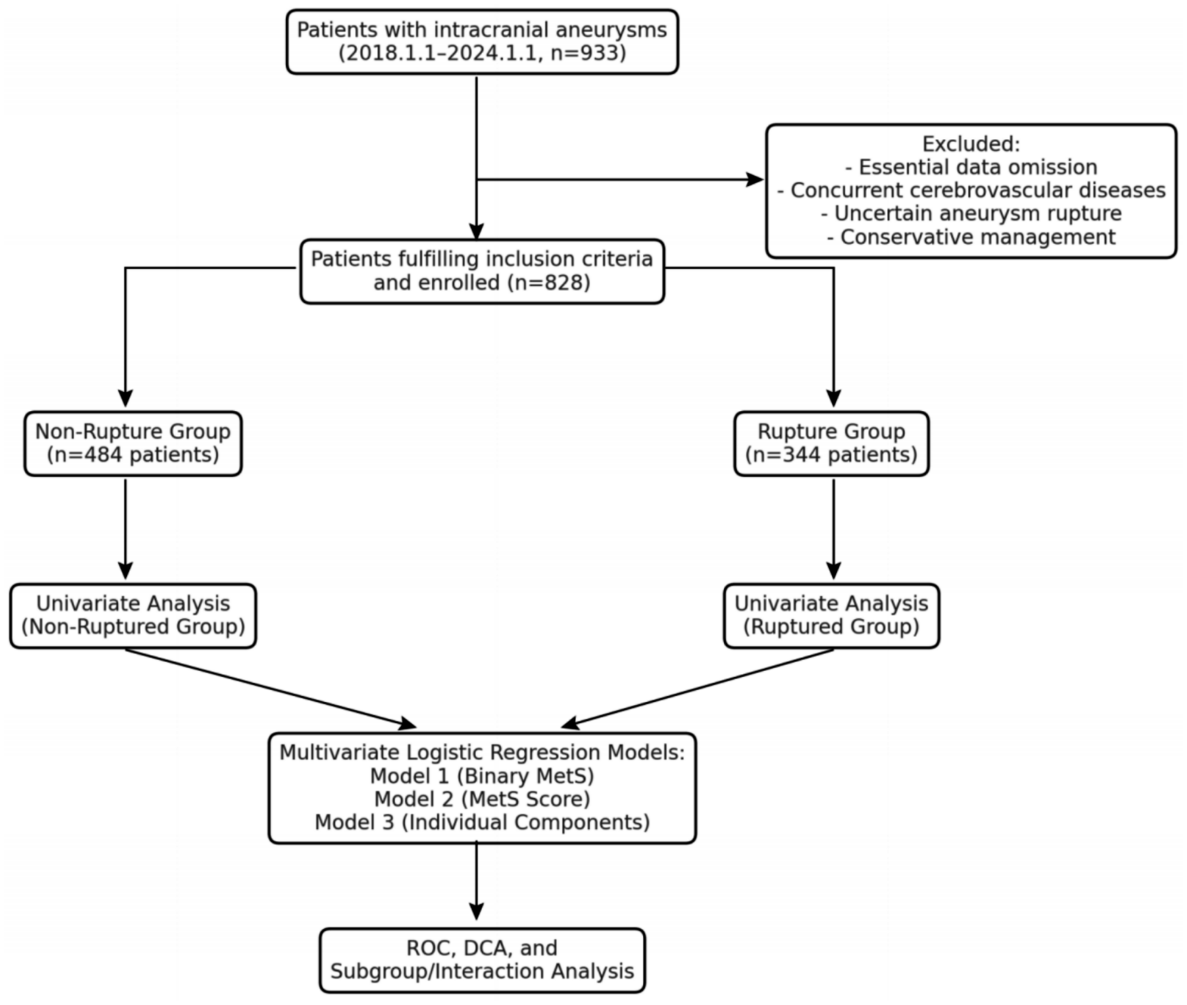
Flowchart illustrating the selection process of patients in the study, detailing the inclusion and exclusion criteria, and the final number of patients included in the analysis.

A total of 828 patients with IAs were included in the study, with 484 patients in the unruptured aneurysm group and 344 in the ruptured aneurysm group. The patient selection process is illustrated in Figure 1. The demographic and clinical characteristics of the study population are presented in Table 2. The mean age of patients in the ruptured aneurysm group was significantly younger than that of the unruptured group (56.40 ± 13.37 years vs. 61.20 ± 11.48 years; *p* < 0.001). The ruptured group had a lower proportion of male patients compared to the unruptured group (45.5% vs. 54.8%; *p* = 0.009). In terms of clinical characteristics, the ruptured aneurysm group exhibited significantly higher SBP (146.23 ± 25.77 vs. 134.80 ± 23.61 mmHg; *p* < 0.001) and DBP (82.39 ± 12.84 vs. 78.89 ± 12.63 mmHg; *p* < 0.001), as well as significantly higher fasting glucose levels (7.72 ± 3.13 vs. 6.82 ± 2.85 mmol/L; *p* < 0.001). However, the ruptured group showed lower rates of hypertension history (48.9% vs. 58.4%; *p* = 0.006) and diabetes history (14.5% vs. 18.3%; *p* < 0.001) compared to the unruptured group. No significant difference was observed in CAD history (5.1% vs. 5.2%; *p* = 0.451). Smoking status and alcohol consumption did not significantly differ between the two groups (*p* = 0.119 and *p* = 0.925, respectively). Interestingly, although the ruptured group demonstrated significantly higher admission SBP and glucose levels, they had lower documented histories of hypertension or diabetes. This discrepancy may reflect a combination of factors, including under-recognition of chronic hypertension or diabetes before rupture, differences in pre-admission medical surveillance between groups, and acute physiological changes after subarachnoid hemorrhage.

**Table 2:** Baseline characteristics stratified by aneurysm rupture status.

Variable	Total (*n* = 828)	Unruptured (*n* = 484)	Ruptured (*n* = 344)	*p*-value
Demographic characteristics				
Age (years)	59.16 ± 12.54	61.20 ± 11.48	56.40 ± 13.37	<0.001
Gender (male), *n* (%)	421 (50.8%)	261 (54.8%)	160 (45.5%)	0.009
BMI (kg/m^2^)	23.79 ± 3.23	23.75 ± 3.28	23.85 ± 3.17	0.309
Lifestyle factors				
Smoking, *n* (%)	168 (20.3%)	106 (22.3%)	62 (17.6%)	0.119
Alcohol consumption, *n* (%)	113 (13.6%)	64 (13.4%)	49 (13.9%)	0.925
Clinical parameters				
SBP (mmHg)	139.66 ± 25.18	134.80 ± 23.61	146.23 ± 25.77	<0.001
DBP (mmHg)	80.38 ± 12.83	78.89 ± 12.63	82.39 ± 12.84	<0.001
Fasting glucose (mmol/L)	7.20 ± 3.00	6.82 ± 2.85	7.72 ± 3.13	<0.001
Triglycerides (mmol/L)	1.61 ± 0.94	1.55 ± 0.80	1.70 ± 1.10	0.719
HDL cholesterol (mmol/L)	1.19 ± 0.28	1.21 ± 0.25	1.17 ± 0.30	0.001
Medical history				
Hypertension history, *n* (%)	450 (54.4)	278 (58.4%)	172 (48.9%)	0.006
DM history, *n* (%)	138 (16.7%)	87 (18.3%)	51 (14.5%)	<0.001
CAD history, *n* (%)	38 (4.6%)	20 (5.2%)	18 (5.1%)	0.451
MetS components				
Elevated BMI, *n* (%)	253 (30.6%)	140 (29.4%)	113 (32.1%)	0.451
Elevated triglycerides, *n* (%)	252 (30.4%)	117 (24.6%)	135 (38.4%)	<0.001
Reduced HDL-C, *n* (%)	360 (43.5%)	178 (37.4%)	182 (51.7%)	<0.001
Elevated BP, *n* (%)	665 (80.3%)	370 (77.7%)	295 (83.8%)	0.037
Elevated glucose, *n* (%)	547 (66.1%)	276 (58.0%)	271 (77.0%)	<0.001
Metabolic syndrome				
MetS score (mean ± SD)	2.51 ± 1.20	2.27 ± 1.15	2.83 ± 1.19	<0.001
MetS, *n* (%)	406 (49.0%)	195 (41.0%)	211 (59.9%)	<0.001

BMI, body mass index; SBP, systolic blood pressure; DBP, diastolic blood pressure; DM, diabetes mellitus; CAD, coronary artery disease; HDL-C, high-density lipoprotein cholesterol; MetS, metabolic syndrome; IQR, interquartile range.

### 3.2 Imaging characteristics in the subsample

**Figure 2:**
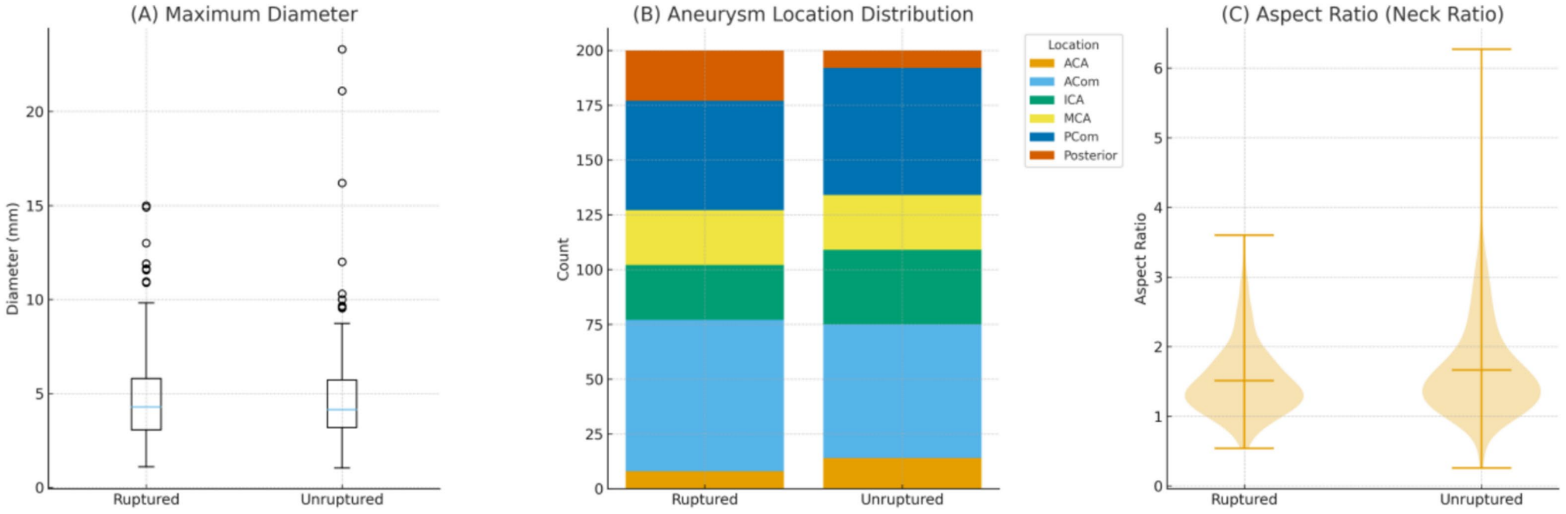
Comparative imaging characteristics between ruptured and unruptured intracranial aneurysms (*n* = 200 per group). **(A)** Boxplot illustrating the distribution of maximum aneurysm diameter for ruptured versus unruptured aneurysms. No significant difference was observed between the two groups (*p = 0*.675). **(B)** Stacked bar chart showing the proportional distribution of aneurysm locations, including internal carotid artery (ICA), posterior communicating artery (PCom), anterior cerebral artery (ACA), anterior communicating artery (ACom), middle cerebral artery (MCA), and posterior circulation. A mild but statistically significant difference in overall location distribution was noted (*p = 0*.045). **(C)** Violin plot comparing the aspect ratio (neck ratio) between ruptured and unruptured aneurysms. The unruptured group demonstrated a slightly higher aspect ratio *p = 0*.047). Mean values are indicated by horizontal bars.

In the imaging subsample of 400 patients (200 ruptured and 200 unruptured), the maximum aneurysm diameter was similar between groups (4.82 ± 2.49 mm vs. 4.71 ± 2.74 mm; *p* = 0.675). Aneurysm location showed a mild overall difference (X2 = 11.1, *p* = 0.045), with ruptured aneurysms more frequently located in the posterior circulation, whereas ICA aneurysms were slightly more common in the unruptured group. The aspect ratio was modestly higher among unruptured aneurysms (*p* = 0.047). Overall, most morphological parameters were comparable, with only subtle differences in location and neck geometry observed between groups (Table 3, Figure 2).

**Table 3:** Comparison of aneurysm morphological characteristics between ruptured and unruptured groups (*n* = 200 per group).

Variable	Ruptured	Unruptured
Maximum diameter, mm (mean ± SD)	4.82 ± 2.49	4.71 ± 2.74
Diameter ≥5 mm, %	40.0	36.5
Irregular aneurysm pattern/cyst, %	43.5	47.5
Bifurcation aneurysm, %	39.0	58.5
Multiple aneurysms, %	18.0	32.5
Wide-neck/carotid aneurysm, %	37.5	36.5

Maximum diameter is presented as mean ± standard deviation (SD).

Diameter ≥5 mm represents the proportion of aneurysms with a maximal diameter of 5 mm or greater.

Irregular aneurysm pattern includes lobulated morphology, daughter sacs, or cyst-like protrusions.

Bifurcation aneurysm refers to lesions originating at arterial bifurcation points, whereas sidewall aneurysms arise along the vessel wall.

Wide-neck/carotid aneurysm was defined according to radiological reports indicating a wide neck or carotid-origin morphology.

No statistically significant differences were observed for maximum diameter or wide-neck morphology between groups (*p* > 0.05).

### 3.3 Univariate analysis of MetS components and rupture

Our study further examined the relationship between MetS and its individual components with aneurysm rupture risk. The results revealed significant differences between the ruptured and unruptured aneurysm groups regarding the positive rates of MetS components. Specifically, elevated BP and elevated glucose were significantly more prevalent in the ruptured group compared to the unruptured group, with rates of 83.8 and 77.0% in the ruptured group, respectively, versus 77.7% and 58.0% in the unruptured group. Additionally, elevated triglycerides (38.4% vs. 24.6%; *p* < 0.001) and reduced HDL-C (51.7% vs. 37.4%; *p* < 0.001) were significantly more common in the ruptured group. In contrast, elevated BMI showed smaller differences between the groups, with positive rates of 32.1% versus 29.4% (*p* = 0.451). Although the difference in mean triglyceride levels between groups was not statistically significant (*p* = 0.719), the proportion of patients exceeding the threshold of 1.7 mmol/L was substantially higher in the ruptured group (38.4% vs. 24.6%, *p* < 0.001), reflecting the fact that elevated TG as a categorical criterion was more informative in group discrimination in distinguishing rupture risk. The prevalence of MetS was higher in the ruptured group (59.9% vs. 40.1%). In addition, higher MetS score categories were more common among ruptured cases, supporting a graded association between greater metabolic burden and ruptured aneurysm status. Table 4 further supports these findings, showing that elevated glucose, elevated triglycerides, elevated blood pressure, and reduced HDL-C were significantly associated with ruptured status in univariable analyses, whereas elevated BMI was not.

**Table 4:** Univariate and multivariate logistic regression analysis of factors associated with IA rupture risk.

Variable	Univariate	Model 1	Model 2	Model 3
Demographic factors				
Age (per year increase)	0.97 (0.96–0.98), <0.001	0.96 (0.95–0.97), <0.001	0.96 (0.95–0.97), <0.001	0.96 (0.95–0.97), <0.001
Gender (female vs. male)	0.69 (0.52–0.91), 0.008	0.65 (0.46–0.91), 0.012	0.61 (0.43–0.86), 0.005	0.49 (0.33–0.71), <0.001
Lifestyle factors				
Smoking (yes vs. no)	0.75 (0.53–1.06), 0.100	0.66 (0.40–1.07), 0.093	0.63 (0.38–1.05), 0.074	0.62 (0.38–1.03), 0.066
Alcohol consumption (yes vs. no)	1.04 (0.70–1.55), 0.844	1.42 (0.83–2.43), 0.195	1.47 (0.86–2.54), 0.163	1.56 (0.90–2.69), 0.111
Clinical parameters				
SBP (per 10 mmHg increase)	1.21 (1.14–1.28), <0.001	1.27 (1.17–1.39), <0.001		
DBP (per 10 mmHg increase)	1.24 (1.11–1.39), <0.001	0.89 (0.76–1.04), 0.138		
Fasting glucose (per 1 mmol/L increase)	1.11 (1.06–1.16), <0.001	1.11 (1.05–1.17), <0.001		
Triglycerides (per 1 mmol/L increase)	1.18 (1.02–1.37), 0.028	0.96 (0.80–1.15), 0.660		
HDL-C (per 0.1 mmol/L increase)	0.94 (0.90–0.99), 0.027	0.97 (0.91–1.03), 0.364		
BMI (per 1 kg/m^2^ increase)	1.01 (0.97–1.05), 0.656	0.95 (0.90–1.00), 0.042		
Medical history				
Hypertension history (yes vs. no)	0.69 (0.53–0.88), 0.024	0.66 (0.47–0.92), 0.014	0.69 (0.50–0.96), 0.027	0.50 (0.35–0.73), <0.001
DM history (yes vs. no)	0.52 (0.35–0.78), 0.006	0.49 (0.31–0.77), 0.002	0.52 (0.33–0.82), 0.003	0.49 (0.32–0.76), 0.001
CAD history (yes vs. no)	1.33 (0.63–2.77), 0. 453	1.35 (0.62–2.65), 0. 433	1.29 (0.65–2.54), 0.468	1.33 (0.63–2.77), 0.453
MetS components				
Elevated BMI (yes vs. no)	1.14 (0.84–1.53), 0.406			0.88 (0.63–1.22), 0.438
Elevated triglycerides (yes vs. no)	1.91 (1.42–2.58), <0.001			1.40 (0.99–1.97), 0.058
Reduced HDL-C (yes vs. no)	1.79 (1.36–2.37), <0.001			2.35 (1.62–3.40), <0.001
Elevated blood pressure (yes vs. no)	1.48 (1.04–2.12), 0.030			1.71 (1.14–2.55), 0.009
Elevated glucose (yes vs. no)	2.42 (1.78–3.30), <0.001			2.56 (1.84–3.56), <0.001
MetS (yes vs. no)	2.16 (1.63–2.86), <0.001	1.83 (1.31–2.54), <0.001	—	—
MetS score (per 1 point increase)				
1	—	—	1.67 (0.65–4.29), 0.290	—
2	—	—	2.02 (0.74–5.53), 0.169	—
3	—	—	2.96 (1.06–8.30), 0.039	—
4	—	—	4.48 (1.50–13.39), 0.007	—
5	—	—	7.57 (2.13–26.92), 0.002	—
Model performance				
AUC	—	0.692	0.697	0.711
Hosmer–Lemeshow χ^2^ (*p*)	—	8.67 (*p* = 0.371)	20.07 (*p* = 0.010)	13.26 (*p* = 0.103)
Nagelkerke *R*^2^	—	0.039	0.042	0.048
Maximum VIF	—	—	—	1.74

Data are presented as odds ratio (95% confidence interval), *p*-value. Model 1 incorporated binary MetS classification (presence vs. absence); Model 2 evaluated MetS severity score (0–5); Model 3 assessed individual MetS components. All multivariate models were adjusted for age, gender, smoking status, alcohol consumption, and relevant comorbidities. AUC, area under the receiver operating characteristic curve; VIF, variance inflation factor.

### 3.4 Multivariate logistic regression

**Figure 3:**
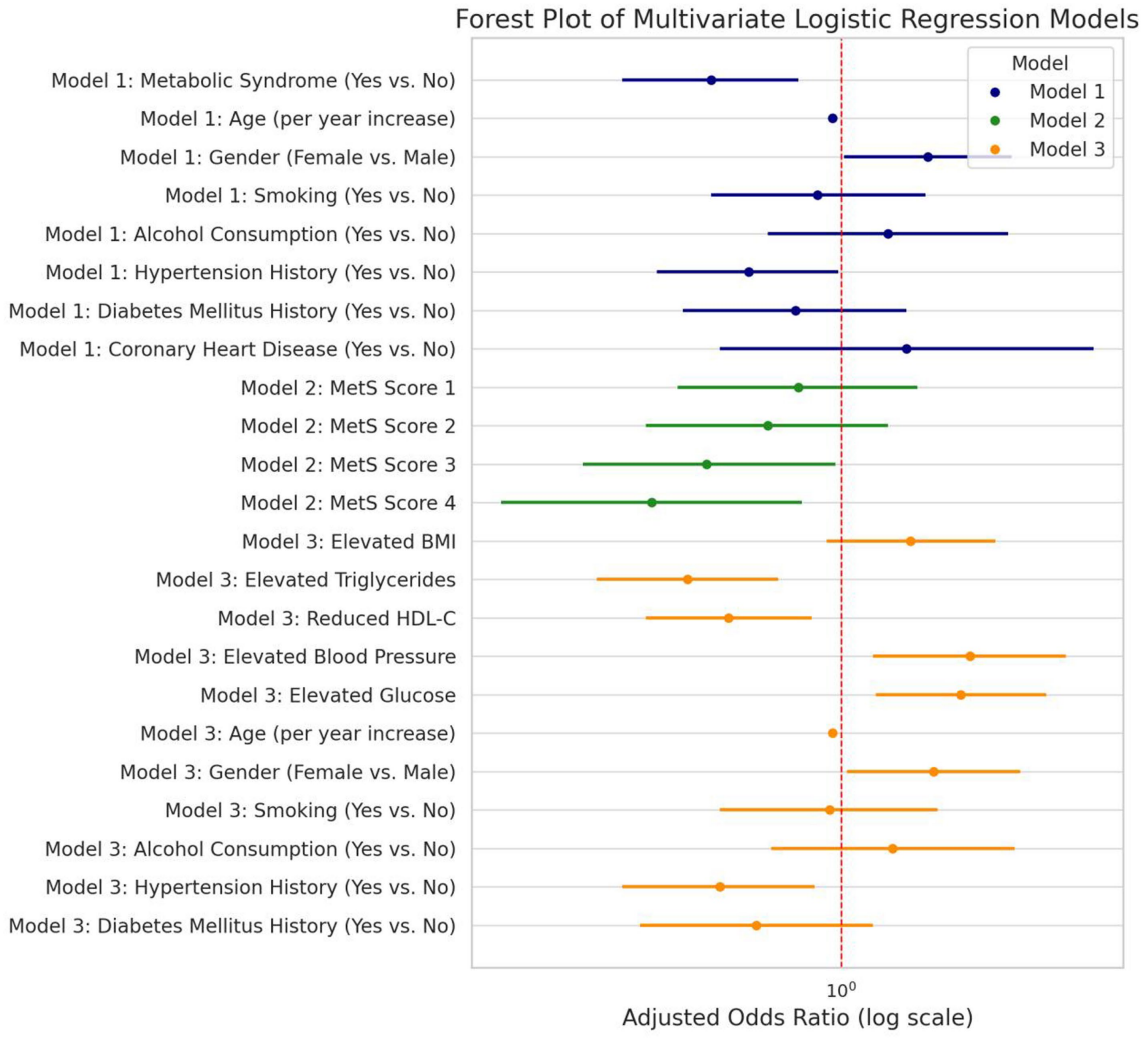
Forest plot comparing three multivariate logistic regression models evaluating metabolic syndrome (MetS) and its components in relation to aneurysm rupture risk. The models include: Model 1: MetS binary classification (Yes vs. No), Model 2: Gradient MetS score effect (reference: score = 0), Model 3: Individual MetS components. Adjusted odds ratios (OR) with 95% confidence intervals are displayed on a log scale, with a vertical dashed red line indicating no association (OR = 1).

Multivariable logistic regression was performed to assess the associations of MetS and its components with ruptured aneurysm status. As shown in Table 4, several metabolic variables, including elevated glucose, elevated blood pressure, reduced HDL-C, and elevated triglycerides, were associated with ruptured status in univariable analyses. In Model 1, binary MetS status was independently associated with ruptured status (adjusted OR 1.83, 95% CI 1.31–2.54). In Model 2, higher MetS scores showed a stepwise association with ruptured status, although the precision of the estimates was lower at the highest score categories. In Model 3, elevated glucose (adjusted OR 2.56, 95% CI 1.84–3.56), reduced HDL-C (adjusted OR 2.35, 95% CI 1.62–3.40), and elevated blood pressure (adjusted OR 1.71, 95% CI 1.14–2.55) showed the strongest independent associations (see Figure 3).

### 3.5 Predictive model performance

**Figure 4:**
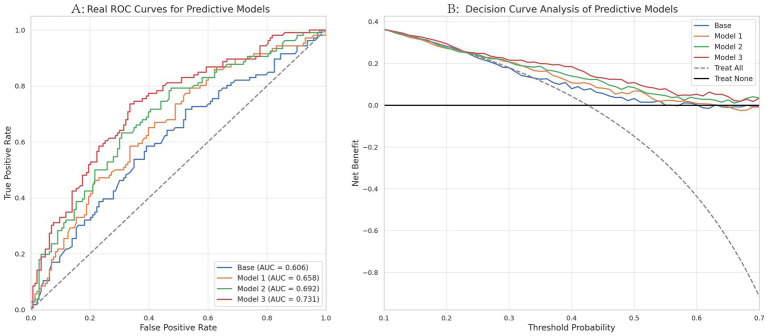
**(A)** ROC curves comparing the discrimination of different models for distinguishing ruptured from unruptured intracranial aneurysms. Areas under the curve (AUC) values are provided in parentheses, with the diagonal dashed line representing random chance performance (AUC = 0.5). **(B)** Decision curve analysis (DCA) showing the net benefit of the base model, MetS binary classification, MetS score, and individual MetS components models across a range of threshold probabilities (0.1–0.7). The curves for “Treat All” and “Treat None” strategies are also shown for reference.

ROC analysis was used to compare the discrimination of the different models for distinguishing ruptured from unruptured aneurysms in this cohort. As shown in Figure 4A, models incorporating MetS-related variables had slightly higher AUC values than the base model. Model 3, which incorporated individual MetS components, showed the highest AUC (0.711), indicating moderate discrimination rather than high predictive accuracy. Figure 4B presents the DCA results, in which Model 3 showed relatively greater net benefit than the comparator models across parts of the examined threshold range. These exploratory findings suggest incremental discriminative value of MetS-related variables within this dataset, but they do not establish definitive exploratory net benefit and require external prospective validation (see Table 5).

**Table 5:** Subgroup analysis of the association between metabolic syndrome and aneurysm rupture.

Subgroup	N	Events (%)	Adjusted OR*	95% CI	*p*-value	P-interaction
Age						0.031
<60 years	407	215 (52.8%)	1.92	1.30–2.85	0.001	
≥60 years	421	137 (32.5%)	2.69	1.76–4.12	<0.001	
Gender						0.028
Male	421	160 (38.0%)	2.47	1.63–3.73	<0.001	
Female	407	192 (47.2%)	2.2	1.48–3.29	<0.001	
Hypertension history						0.483
Yes	450	172 (38.2%)	2.19	1.46–3.26	<0.001	
No	378	180 (47.6%)	2.71	1.77–4.15	<0.001	
DM history						0.019
Yes	138	51 (37.0%)	1.99	0.91–4.33	0.084	
No	690	301 (43.6%)	2.35	1.72–3.19	<0.001	
CAD history						0.752
Yes	38	18 (47.4%)	1.11	0.27–4.55	0.88	
No	790	334 (42.3%)	2.21	1.66–2.95	<0.001	

Adjusted for age, gender, smoking, alcohol consumption, hypertension history, diabetes history, and coronary artery disease, as appropriate for each subgroup analysis.

### 3.6 Subgroup and interaction analyses

**Figure 5:**
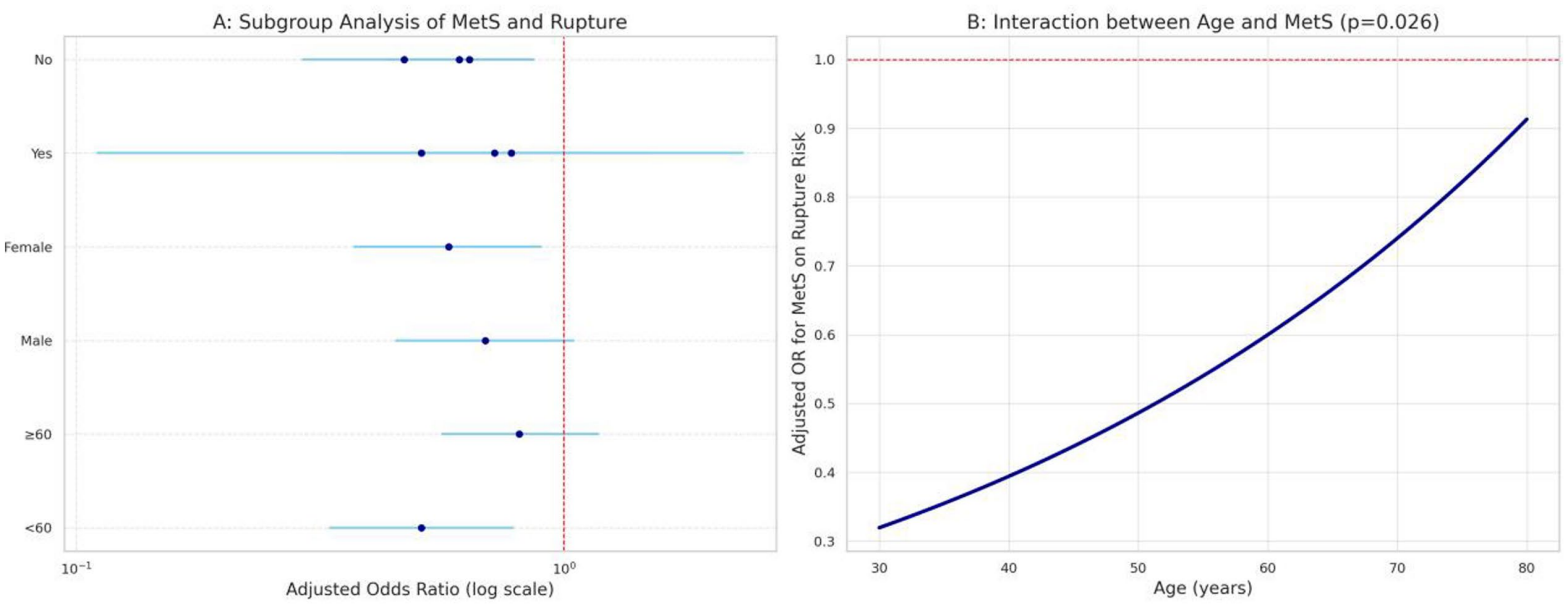
**(A)** Forest plot illustrating subgroup analysis of the association between metabolic syndrome (MetS) and ruptured aneurysm status stratified by age (<60 vs. ≥ 60 years), gender (male vs. female), hypertension history, and diabetes mellitus history. Adjusted odds ratios (OR) with 95% confidence intervals (CIs) are presented on a logarithmic scale, with a vertical dashed line indicating no effect (OR = 1). *p*-values for interaction between subgroups are included where applicable. **(B)** Interaction plot demonstrating how the adjusted odds ratio for ruptured status associated with MetS varies across different ages. The blue curve represents the estimated OR across ages 30 to 80 years, derived from the interaction term between age and MetS. The horizontal dashed red line (OR = 1) indicates no association, and the significance of the interaction effect (*p*-value) is explicitly stated within the plot title.

This analysis explored whether the association between MetS and ruptured aneurysm status varied across selected subgroups. Figure 5A shows that the association remained present across age, sex, hypertension-history, and diabetes-history strata, with significant interaction by age and diabetes history. Figure 5B suggests that the association between MetS and ruptured status became more pronounced with increasing age. These findings should be interpreted cautiously as exploratory subgroup results.

### 3.7 Morphology-adjusted sensitivity analyses

In the imaging subsample with available aneurysm morphology data (*n* = 400), the association between MetS and ruptured aneurysm status remained broadly similar after additional adjustment for morphology-related variables. Binary MetS was associated with ruptured status after clinical adjustment alone (OR 3.40, 95% CI 2.23–5.18, *p* < 0.001), and this estimate was not meaningfully changed after further adjustment for aneurysm irregularity, multiplicity, grouped location, bifurcation/sidewall type, maximum diameter, and neck ratio (OR 3.76, 95% CI 2.36–5.99, *p* < 0.001). Likewise, each 1-point increase in MetS score remained associated with ruptured status before (OR 1.82, 95% CI 1.50–2.21, *p* < 0.001) and after morphology adjustment (OR 1.89, 95% CI 1.52–2.33, *p* < 0.001). These results support the robustness of the main association within this imaging subset.

## 4 Discussion

### 4.1 Key findings and interpretation

In this treated retrospective cohort, MetS was independently associated with ruptured IA status. Patients meeting MetS criteria had higher odds of belonging to the ruptured group, and higher MetS score categories showed a graded association with ruptured status. Among the individual components, elevated blood pressure, elevated glucose, and reduced HDL-C showed the strongest independent associations. These findings suggest that metabolic dysregulation may be relevant to aneurysm instability, although the retrospective design and the timing of measurements in the ruptured group preclude causal inference. The subgroup analyses further suggested that these associations may vary across patient strata, particularly by age, but these exploratory findings require cautious interpretation. MetS-related variables also provided modest incremental discrimination within this dataset, although the magnitude of improvement was limited.

### 4.2 Plausible biological mechanisms linking MetS to aneurysm rupture

The link between MetS and aneurysm rupture is biologically plausible, given MetS’s well-documented effects on the vasculature (11). MetS comprises interrelated factors—hypertension, impaired glucose regulation, dyslipidemia, and central obesity—that collectively foster chronic low-grade inflammation and endothelial dysfunction (12). Prolonged hypertension, a core feature of MetS, imposes persistent hemodynamic stress on arterial bifurcations, promoting endothelial cell turnover and inflammatory cell infiltration. These processes facilitate extracellular matrix (ECM) degradation, smooth muscle cell loss, and overall weakening of the vessel wall, setting the stage for aneurysm formation and eventual rupture.

An intriguing observation in our findings is that, despite a lower prevalence of documented hypertension history in the ruptured group, elevated SBP at presentation strongly correlates with increased rupture risk. This discrepancy likely reflects variations in hypertension detection and treatment between the groups: patients with established hypertension in the unruptured cohort often receive antihypertensive therapy (e.g., ACE inhibitors or calcium-channel blockers), resulting in more effective BP control and reduced rupture susceptibility (13, 14). Conversely, individuals in the ruptured group may harbor undiagnosed or suboptimally managed hypertension, manifesting as markedly elevated SBP during rupture (15, 16). Collectively, these findings underscore the importance of distinguishing well-controlled, longstanding hypertension from acute SBP surges when evaluating aneurysm rupture risk (17). This pattern should be interpreted cautiously. It may reflect underdiagnosis or undertreatment of chronic hypertension before rupture, differences in medical surveillance between treated unruptured and ruptured patients, and/or acute post-hemorrhagic blood pressure elevation. Accordingly, the admission BP findings in this study should not be interpreted as direct evidence of pre-rupture long-term blood pressure exposure.

A similar interpretive caveat applies to glucose measurements in the ruptured group. Hyperglycemia and insulin resistance further heighten vascular injury through multiple pathways. Elevated glucose levels promote oxidative stress by triggering excessive production of reactive oxygen species, which directly damage endothelial cells and ECM proteins (18). Concurrently, advanced glycation end-products stiffen arterial walls and reduce their elasticity (19). Insulin resistance also drives a pro-inflammatory, prothrombotic milieu characterized by elevated IL-6 and TNF-*α*, impairing the vasa vasorum and hindering normal repair mechanisms (20). These insults can manifest as a degenerative, inflamed IAs wall that is less capable of withstanding hemodynamic loads.

Dyslipidemia—particularly low HDL-cholesterol—represents another key driver. HDL normally confers anti-inflammatory and antioxidant benefits, aiding endothelial repair and nitric oxide production (21, 22). In its absence, inflammatory and oxidative cascades can accelerate wall damage. MetS often features atherogenic lipid profiles (e.g., small dense LDL, postprandial lipemia), which may infiltrate cerebral arterial walls and instigate local inflammation (23, 24). Although some studies suggest hypercholesterolemia or diabetes alone could correlate with reduced rupture risk—possibly through arterial wall thickening or protective remodeling—our data and the broader literature highlight that, within MetS, the net effect of poor lipid profiles (low HDL and high triglycerides) is detrimental.

Ultimately, MetS primes the aneurysm wall for rupture through synergistic mechanisms: chronic inflammation, oxidative stress, endothelial dysfunction, and maladaptive vascular remodeling. Histopathological findings in ruptured IAs, such as intense inflammatory infiltrates and marked ECM degeneration, further support these processes. The cumulative effect is an arterial wall ill-equipped to tolerate normal pulsatile forces, making MetS a potent contributor to aneurysm instability.

### 4.3 Comparison with previous studies in vascular diseases

Our findings build on extensive evidence linking MetS to adverse vascular outcomes. Initially conceptualized in a cardiovascular context, MetS was shown to double the risk of cardiovascular death in a prospective study of middle-aged men (11). Similarly, in a 20-year follow-up of initially healthy individuals, those with MetS experienced higher rates of stroke and myocardial infarction than those without MetS (25). These broad observations align with our conclusion that MetS contributes not only to atherothrombotic events but also to hemorrhagic complications such as IA rupture.

Beyond the cerebral circulation, MetS has been associated with more aggressive aneurysmal changes in abdominal aortic aneurysm (AAA) disease, including larger diameters and higher rupture rates (26). This parallels our findings in IAs and suggests a common pathophysiologic mechanism involving inflammation and vessel wall remodeling. However, a notable difference arises with diabetes: while diabetes mellitus inversely correlates with AAA incidence and progression—possibly due to increased collagen crosslinking or atherosclerotic plaques stabilizing the arterial wall—our study indicates that in the cerebral circulation, hyperglycemia and insulin resistance are deleterious (27, 28). Such discrepancies may reflect distinct vascular bed responses or varying durations and management of diabetes. Indeed, certain cerebrovascular studies show that tight glycemic control can reduce subarachnoid hemorrhage risk, whereas poorly controlled diabetes raises rupture risk (29). Despite occasional paradoxical findings, the overall picture reinforces the importance of metabolic control in safeguarding vascular integrity.

Parallel patterns also emerge in CAD. MetS remains an independent predictor of recurrent cardiac events and mortality, even if BP or LDL-cholesterol levels are managed to guideline targets (30). Consistent with that, we found the composite MetS status (or high MetS scores) was more predictive of IA rupture than any single component. Low HDL-cholesterol, a staple of MetS-related dyslipidemia, is particularly problematic: although some studies suggest hypercholesterolemia may reduce aneurysm rupture risk (a phenomenon analogous to the “diabetes paradox”) (31), our results emphasize that a classic atherogenic profile—low HDL and high triglycerides—amplifies aneurysm vulnerability. This distinction reflects differences between atherosclerotic occlusion and aneurysm formation, where systemic risk factors can exert complex, and sometimes conflicting, influences.

Overall, our data echo the broader observation that MetS exacerbates vascular damage across multiple arterial territories (32, 33). In stroke research, for instance, MetS markedly increases both ischemic and hemorrhagic stroke incidence, and is found in nearly half of patients with stroke or TIA—substantially above population averages (25, 32, 34). Our findings now extend this trend to aneurysmal subarachnoid hemorrhage, underscoring that metabolic risk factor clustering can accelerate cerebrovascular pathology. By comparing IA rupture with AAA, CAD, and other systemic vascular conditions, we highlight a consistent pattern wherein MetS fosters arterial instability, yet acknowledge unique nuances (like the diabetes and lipid paradoxes) that warrant further mechanistic and clinical exploration.

### 4.4 Clinical implications

The present findings do not support immediate clinical implementation. Rather, they suggest that metabolic profiling may provide complementary information when interpreting ruptured versus unruptured status in treated cohorts and may help generate hypotheses for future prospective studies. Whether aggressive management of hypertension, dysglycemia, and dyslipidemia can influence aneurysm growth, instability, or rupture-related outcomes remains uncertain and should be addressed in longitudinal research.

### 4.5 Limitations

Several limitations merit consideration. First, this was a retrospective single-center study, which limits generalizability and is vulnerable to selection bias. In particular, because only treated unruptured aneurysms were included and conservatively managed cases were excluded, the study cohort does not represent the full natural-history spectrum of unruptured IAs. Second, although an imaging subsample analysis was performed, standardized aneurysm morphology data were unavailable for the full cohort; therefore, residual confounding by size, location, and other rupture-related morphological features cannot be excluded. Third, blood pressure and glucose in the ruptured group were measured after subarachnoid hemorrhage, and these admission values may partly reflect acute stress physiology rather than pre-rupture long-term metabolic status. Fourth, although smoking and alcohol consumption were adjusted for in the multivariable models, some potentially relevant confounders, including family history and detailed medication exposure, were not systematically available. Finally, the observed AUC values indicate only moderate discrimination, and the exploratory DCA findings should not be interpreted as proof of immediate exploratory net benefit.

### 4.6 Future directions

Future prospective, multicenter studies are needed to determine whether MetS and its components are longitudinally associated with aneurysm growth, instability, and rupture-related outcomes. Longitudinal studies that track patients with unruptured aneurysms and monitor their metabolic health over time will be crucial for understanding the dynamics of MetS in aneurysm progression. Additionally, controlled trials focusing on the modification of metabolic risk factors—through antihypertensive, glucose-lowering, and lipid-modulating therapies—are needed to assess whether these interventions can reduce rupture rates and improve patient outcomes.

Mechanistic studies using animal models of aneurysm formation, or molecular analyses of human aneurysmal tissue, could reveal the exact pathways by which MetS influences aneurysm wall degeneration (35). Identifying specific biomarkers or molecular mechanisms (such as inflammatory signaling or matrix metalloproteinase activity) could inform the development of targeted therapeutic approaches. Drugs aimed at reducing chronic inflammation or improving insulin sensitivity—such as IL-1β inhibitors or metformin—should be explored in future trials for their potential to stabilize aneurysm walls.

## 5 Conclusion

In this single-center retrospective study of a treated IA cohort, MetS and greater MetS burden were associated with ruptured aneurysm status, with stronger associations observed for elevated blood pressure, elevated glucose, and reduced HDL-C. These findings suggest that metabolic dysfunction may be relevant to aneurysm instability in treated cohorts. However, because of the retrospective design, treated-cohort selection, incomplete morphology data, and the possibility that acute post-rupture physiology influenced admission measurements, the results should be interpreted as associative and hypothesis-generating rather than causal or immediately practice-changing. Future prospective multicenter studies are needed to validate these associations.

## Data Availability

The datasets presented in this article are not readily available because the data sets generated during the current study are available from the corresponding author on reasonable request. Requests to access the datasets should be directed to liuzishengcc@163.com.
