# Supplementary material for: Association of metabolic syndrome with ruptured status of intracranial aneurysms in a definitively treated cohort: a retrospective cohort analysis
Source: Front Neurol. 2026 May 22;17:1817370. doi: 10.3389/fneur.2026.1817370 (PMC13236563; doi:10.3389/fneur.2026.1817370)
Supplement: Supplementary file 1 [file Data_Sheet_1.zip › supply Figure/Supply Figure legend.docx]

**Figure 1:**Violin plots displaying the distribution and comparison of metabolic characteristics (BMI, blood glucose, triglycerides, HDL cholesterol, systolic blood pressure, and diastolic blood pressure) between the ruptured and unruptured aneurysm groups. The width of each violin indicates data density, and embedded box plots show the median and interquartile range.

**Figure 2:**
Bar plots illustrating the distribution of clinical characteristics (gender, smoking, alcohol consumption, hypertension history, diabetes history, and coronary heart disease history) between the ruptured and unruptured aneurysm groups.

**Figure 3:**Bar charts comparing the positive rates (%) of five components of metabolic syndrome (Elevated BMI, Elevated TG, Reduced HDL-C, Elevated BP, Elevated Glucose) between the ruptured and unruptured intracranial aneurysm groups.

**Figure 4:**(A) Stacked bar chart illustrating the percentage of patients meeting the diagnostic criteria for Metabolic Syndrome (MetS) in ruptured and unruptured intracranial aneurysm groups.
(B) Stacked bar chart showing the distribution of MetS scores (ranging from 0 to 5) by aneurysm rupture status, revealing trends in rupture likelihood with increasing MetS severity.

**Figure 5:**
Forest plot showing univariate logistic regression analysis, demonstrating the association between demographic, clinical, and metabolic variables with aneurysm rupture risk. Odds ratios (OR) are plotted on a log scale with 95% confidence intervals, with a vertical dashed red line indicating no effect (OR=1).
