# Supplementary figures and images for: Association of metabolic syndrome with ruptured status of intracranial aneurysms in a definitively treated cohort: a retrospective cohort analysis
Source: Front Neurol. 2026 May 22;17:1817370. doi: 10.3389/fneur.2026.1817370 (PMC13236563; doi:10.3389/fneur.2026.1817370)

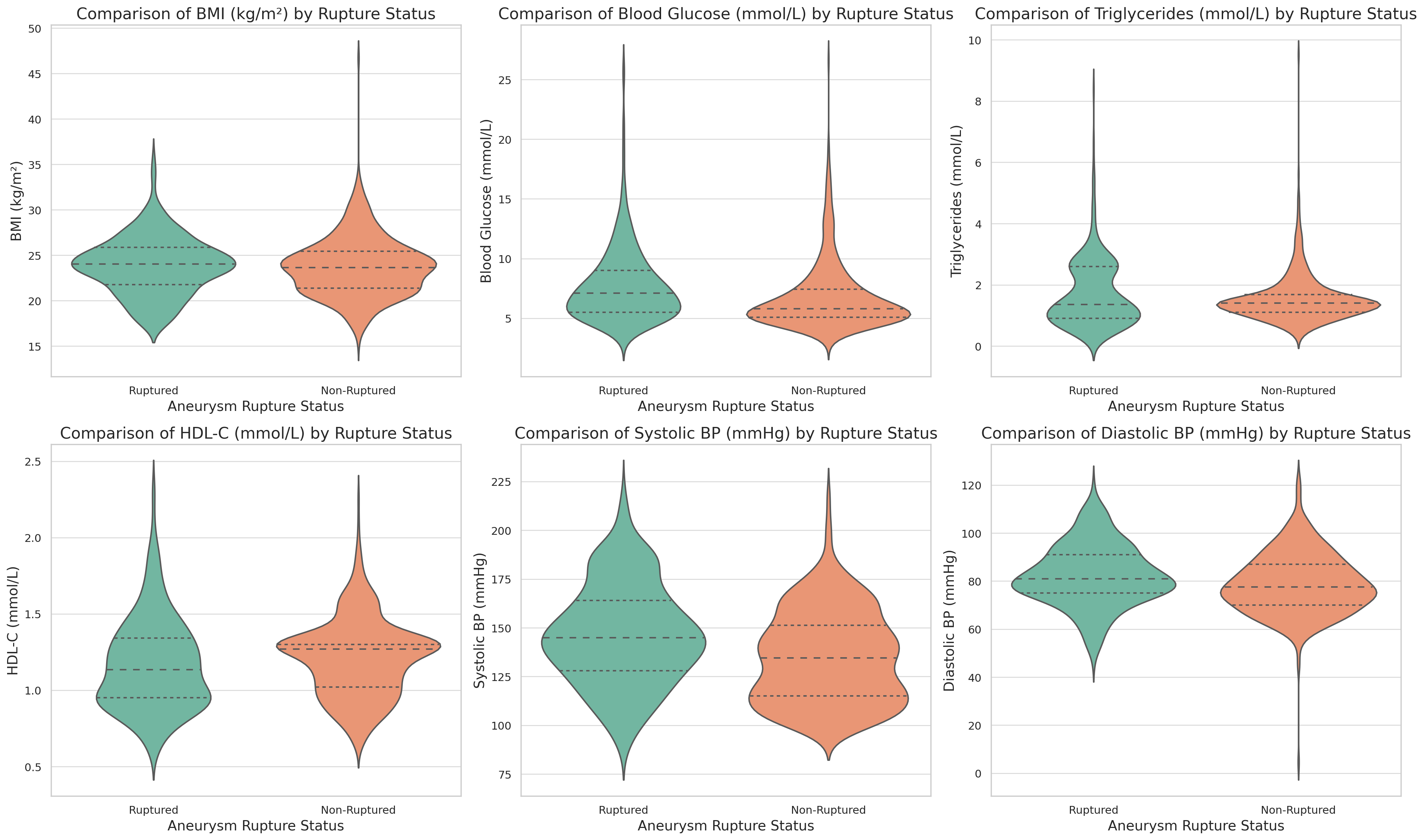

Supplement: Supplementary file 1 [file Data_Sheet_1.zip › supply Figure/supply Figure1.png]

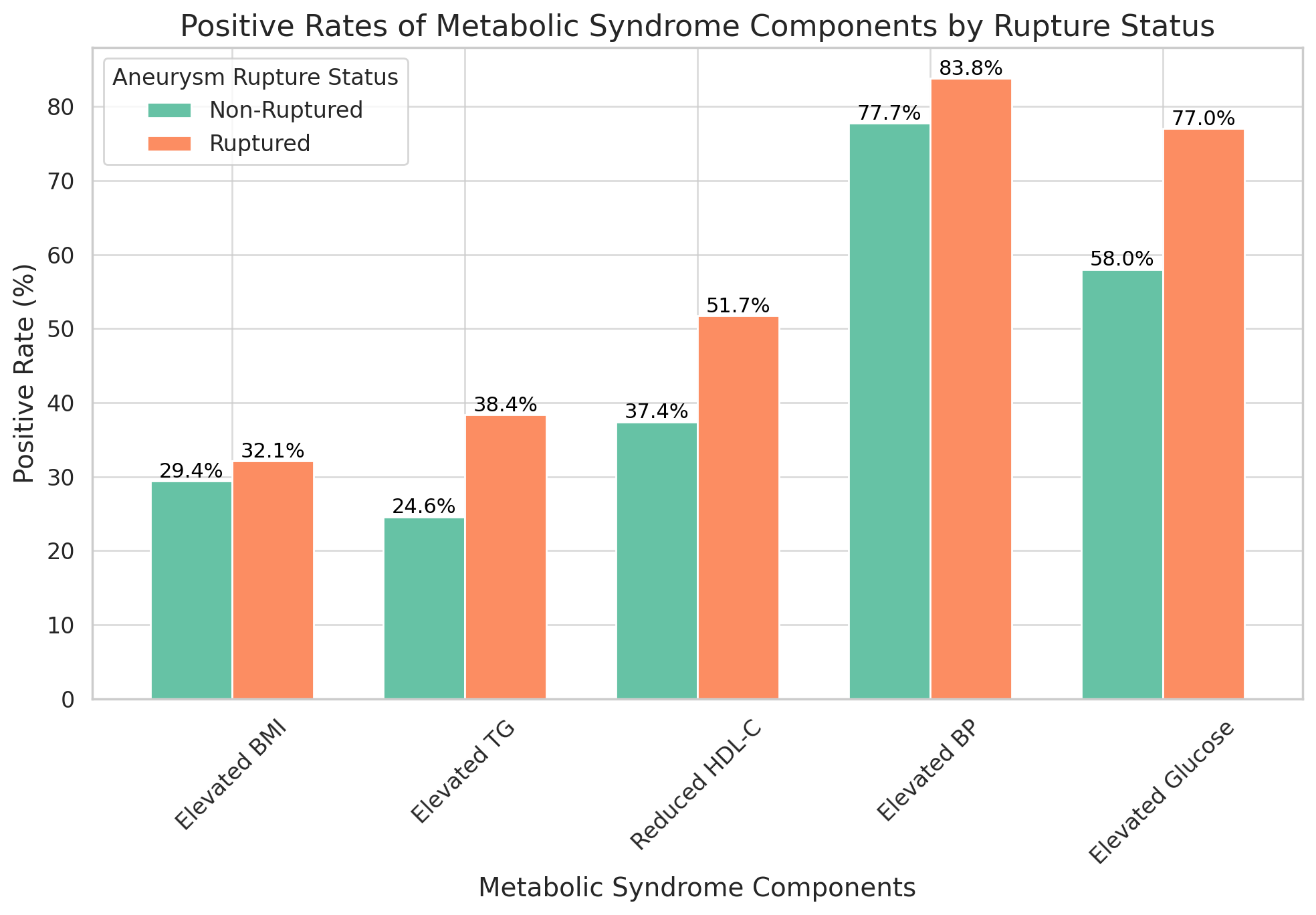

Supplement: Supplementary file 1 [file Data_Sheet_1.zip › supply Figure/supply Figure3.png]

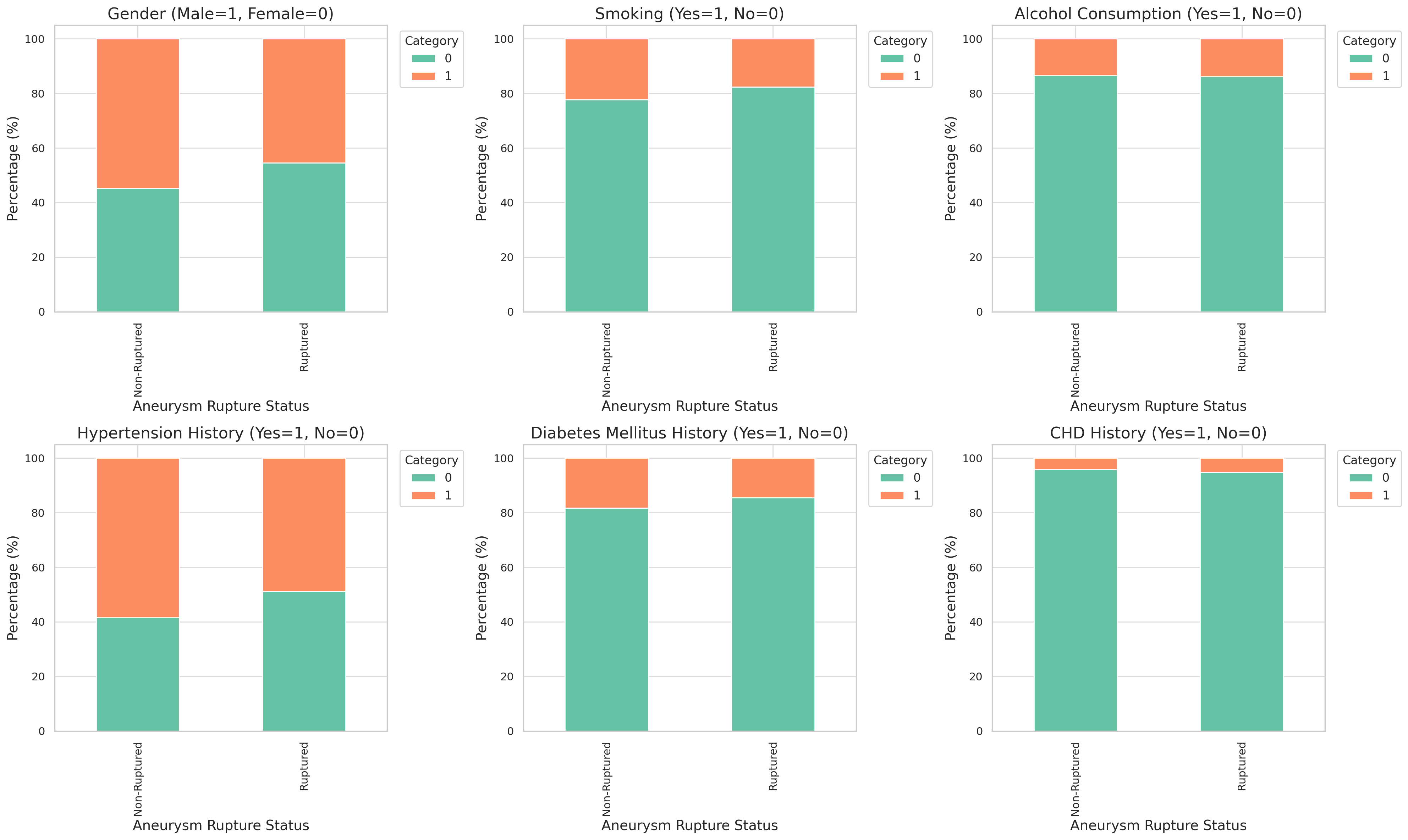

Supplement: Supplementary file 1 [file Data_Sheet_1.zip › supply Figure/supply Figure2.png]

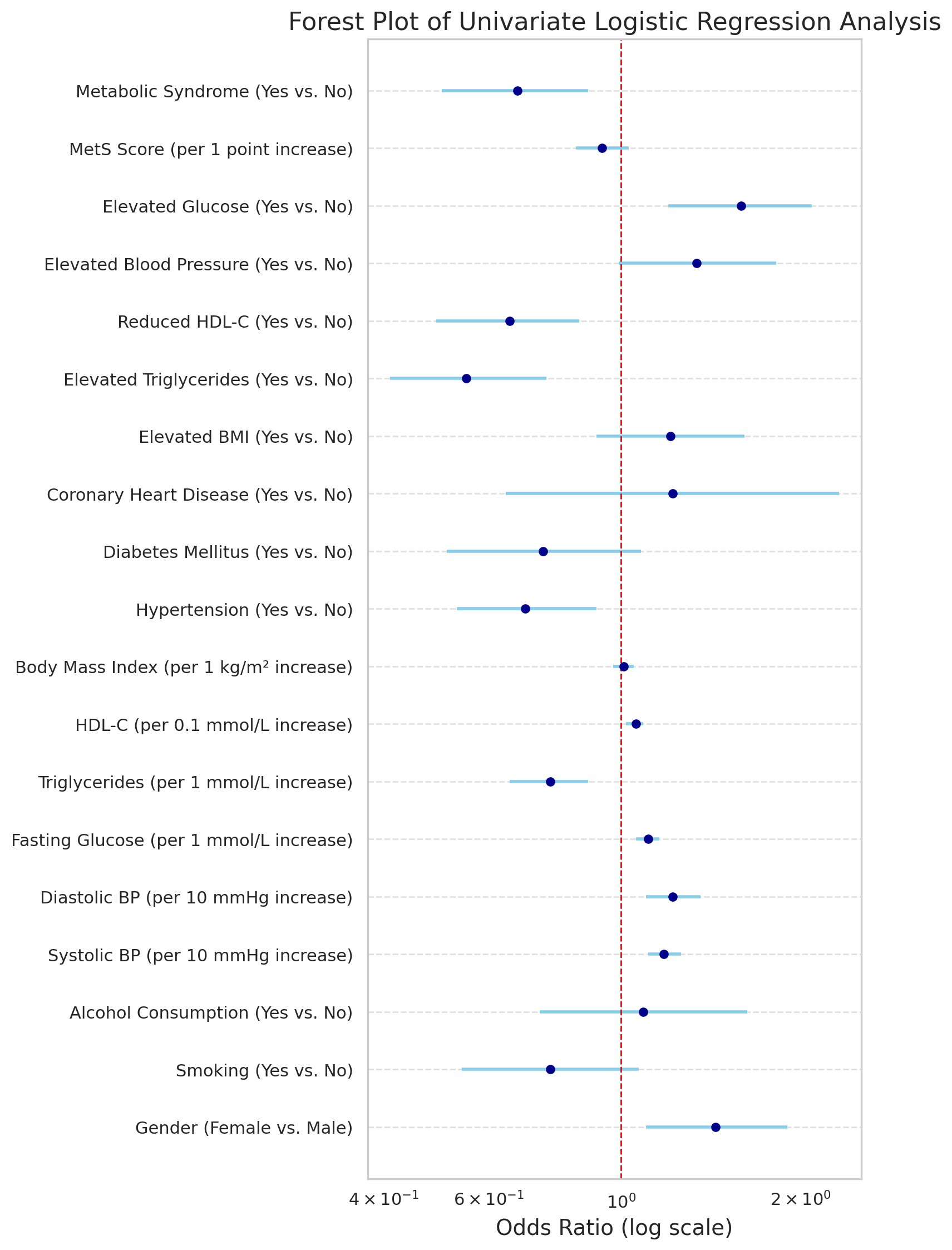

Supplement: Supplementary file 1 [file Data_Sheet_1.zip › supply Figure/supply Figure5.png]

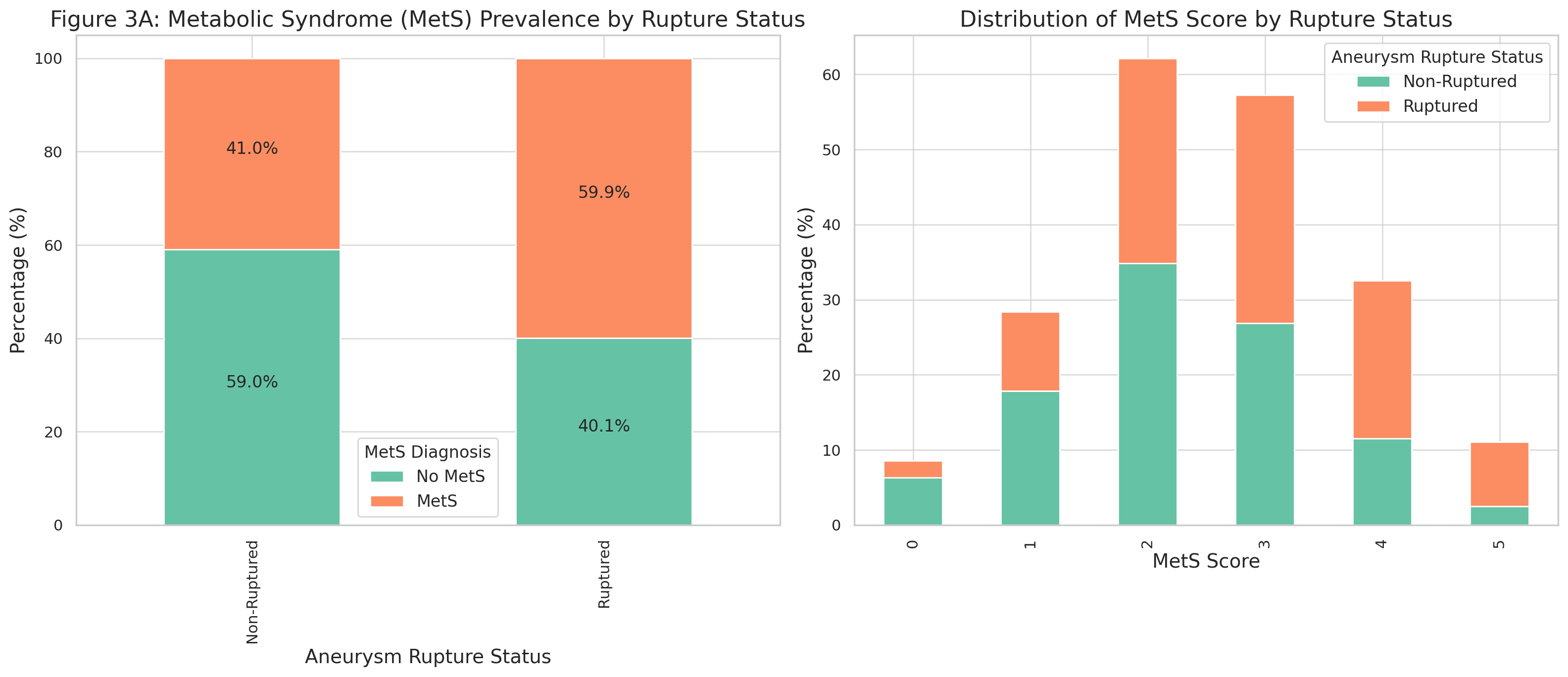

Supplement: Supplementary file 1 [file Data_Sheet_1.zip › supply Figure/supply Figure4.png]
